# Transcriptome analysis on responses of orchardgrass (*Dactylis glomerata* L.) leaves to a short term flooding

**DOI:** 10.1186/s41065-020-00134-0

**Published:** 2020-05-17

**Authors:** Dandan Qiao, Yajie Zhang, Xuemei Xiong, Mingyang Li, Kai Cai, Hui Luo, Bing Zeng

**Affiliations:** College of Animal Science, Southwest University, Rongchang District, Chongqing, 402460 China

**Keywords:** Orchardgrass, Flooding, Transcriptome profiling, ROS scavenging

## Abstract

**Background:**

Orchardgrass (*Dactylis glomerata* L.) is a popular cool-season perennial grass with a high production value, and orchardgrass seed is the fourth top-selling forage grass seed in the world. However, its yield and quality are often affected by flooding. To date, the molecular responses of orchardgrass to flooding were poorly understood.

**Results:**

Here, we performed mRNA-seq to explore the transcriptomic responses of orchardgrass to a short term flooding (8 h and 24 h). There were 1454 and 565 differentially expressed genes identified in the 8 h and 24 h of flooding, respectively, compared to well control. GO functional enrichment analysis showed that oxidoreductase activity and oxidation-reduction process were highly present, suggesting that flooding induced the response to oxygen stress. Pathways enrichment analysis highlights the importance of glutathione metabolism, peroxidase, glycolysis and plant hormone signal transduction in response to flooding acclimation. Besides, the ROS clearance system is activated by significantly expressed glutathione S-transferase and genes encoding SOD and CAT (*CAT1* and *CDS2*). The significant positive correlation between RNA sequencing data and a qPCR analysis indicated that the identified genes were credible.

**Conclusion:**

In the process of orchardgrass response to flooding stress, multiple differential genes and biological processes have participated in its acclimation to flooding, especially the biological processes involved in the removal of ROS. These results provide a basis for further research on the adaptation mechanism of orchardgrass to flood tolerance.

## Background

Flooding is a major factor, limiting growth and production of plants on a global scale [1]. Agricultural development is hindered by flooding every year in many countries and regions around the world. For example, in 2012, flooding were devastating to economic development, and many indigenous fisheries and farming communities in the delta were damaged due to excess water covered in the area (Nigeria) [2]. In 2018, 24 provinces, autonomous regions, and municipalities suffered from floods in China. The area of crops affected by disasters reached 1304 thousand hectares, and the direct economic loss was up to 25.9 billion RMB. Therefore, flooding has considerably threatened the food security of developing countries and even has become a challenge for agricultural development in all countries of the world.

Plants suffer from various biotic and abiotic stresses throughout their life span. Higher plants are aerobic organisms. Flood is a kind of abiotic stress which have a devastating impact on crop growth and survival and thus on food production [3]. The main damage caused by flooding to plants is insufficient root oxygen supply caused by the poor gas exchange, affecting the synthesis of ATP and plant metabolism [4]. In addition, flood can also lead to plant mineral deficiency, nutrient imbalance or changes in nutrient distribution [5]. The physiological disturbances of plant roots caused by flooding can alter the concentration of macroscopic and trace elements in endogenous substances while changing the absorption of nutrients [6]. flooding can cause a variety of physiological disorders that affect plant growth, including root water flux reduction, hormone imbalance, carbohydrate distribution changes, nutrient absorption, early leaf senescence, and organ damage [7].

RNA-seq technology has higher sensitivity to capture low and high-level gene expression than array technologies [8]. RNA-seq can not only detect information about the relationship between a gene and its products, but also isolate genes that are difficult to handle by biochemistry [9]. The technique has been widely used to identify key pathways and differential gene expression in a variety of plant species involved in regulation during abiotic stress, such as drought stress [10], salt stress [11], heat stress [12], cold stress [13]. Recently, the use of transcriptome sequencing technology to study the response of plants to flooding stress is endless. Studies in *Zea mays* [14], *Nymphoides peltata* [15], *Cucumis sativus* [16], *Persea americana* [17], *Sesamum indie* [18], *Taxodium* [19], *Cerasus sachalinensis* [20] have revealed that antioxidative processes, carbohydrates, photosynthesis, glycolysis, phytohormone signal transduction, transcription factors (*ERFs, MYB, HSP,* and *MAPK*) and other metabolic processes are involved in the domestication of plants for flooding stress and play an important role. The results provide a better understanding for the adaptation mechanism of plants to flooding stress.

Orchardgrass (*Dactylis glomerata* L.) is one of the most important cool-season forage grasses [21]. Due to its high sugar and protein content, large biomass, and strong shade tolerance, it has been grown in East Asia, Europe, and North America for more than 100 years [22]. Orchardgrass seed is the fourth top-selling forage grass seed in the world [23]. It is widely planted in southwestern China for green feeding, hay or silage due to its strong adaptability and good palatability. In recent years, the research on orchardgrass has been increasing, and most of the research on orchardgrass resistance mainly focuses on drought stress [24], heat stress [25], and rust stress [26]. However, little is known about the flooding tolerance of orchardgrass and the transcriptome response of this species to flooding stress has not been reported. The objectives of this study were to identify the differentially expressed genes and analyze related pathways from transcriptome sequencing of orchardgrass. The results will provide a reference for further studying the molecular and genetic mechanisms of flooding tolerance in orchardgrass and other related perennial grass species.

## Results

### Transcriptome sequencing and mapping of Illumina reads

To comprehensively clarify the flooding tolerance of orchardgrass, the plants of orchardgrass seedlings were flooded at different times (0 h (CK), 8 h and 24 h). Sequencing results obtained raw data 427,211,572 and the raw reads were filtered obtain clean reads was 416,737,748 (Table S1). The orchardgrass genome was used as the reference genome. Approximately72% of the clean reads were total mapped to the reference genome, with more than 69% of them being uniquely mapped (Table 1). The raw data of this study was stored in the NCBI (National Coalition Building Institute) SAR (Sequence Read Archive) database, which can be viewed in BioProject (ID: PRJNA554779).

**Table 1:** Analysis of statistical data RNA-seq of orchardgrass

Sample^a^	WS_0h_1	WS_0h_2	WS_0h_3	WS_8h_1	WS_8h_2	WS_8h_3	WS_24h_1	WS_24h_2	WS_24h_3
Total reads^b^	44,643,436	51,100,544	41,539,096	48,916,078	46,175,616	39,333,770	39,625,568	48,857,684	56,545,956
Total mapped (%)^c^	32,213,341	36,722,643	29,484,126	34,653,721	32,228,040	27,919,523	28,219,174	33,577,514	39,533,008
	(72.16%)	(71.86%)	(70.98%)	(70.84%)	(69.79%)	(70.98%)	(71.21%)	(68.73%)	(69.91%)
Multiple mapped (%)^d^	1,120,609	1,233,315	946,216	1,225,851	1,073,156	899,453	852,050	1,147,239	1,276,249
	(2.51%)	(2.41%)	(2.28%)	(2.51%)	(2.32%)	(2.29%)	(2.15%)	(2.35%)	(2.26%)
Uniquely mapped (%)^e^	31,092,732	35,489,328	28,537,910	33,427,870	31,154,884	27,020,070	27,367,124	32,430,275	38,256,759
	(69.65%)	(69.45%)	(68.7%)	(68.34%)	(67.47%)	(68.69%)	(69.06%)	(66.38%)	(67.66%)
Reads map to ‘+’ (%)^f^	15,563,768	17,748,957	14,279,174	16,684,717	15,561,283	13,493,851	13,693,019	16,209,484	19,136,212
	(34.86%)	(34.73%)	(34.38%)	(34.11%)	(33.7%)	(34.31%)	(34.56%)	(33.18%)	(33.84%)
Reads map to ‘-’ (%)^g^	15,528,964	17,740,371	14,258,736	16,743,153	15,593,601	13,526,219	13,674,105	16,220,791	19,120,547
	(34.78%)	(34.72%)	(34.33%)	(34.23%)	(33.77%)	(34.39%)	(34.51%)	(33.2%)	(33.81%)

^a^WS_0h_1, 2, 3 (flooding treated for 0 h), WS_8h_1, 2, 3 (flooding treated for 8 h), WS_24h_1, 2, 3 (flooding treated for 24 h); ^b^The amount of RNA-seq sequencing sequence filtered by sequencing data (Clean data);^c^Total Mapped Reads and their percentage of clean reads; ^d^Multiple Mapped Reads and their percentage of clean reads; ^e^Uniquely Mapped Reads and their percentage of clean reads; ^f, g^Sequencing sequence alignment to the total number of positive and negative strands on the genome (Total Mapped Reads) and its percentage of clean reads

Pearson correlation analysis was performed to validate the gene expression profiles of transcripts from nine different samples (Figure S1). The results showed that biological replicates from the same treatment group were highly correlated. In particular, ws_0h and ws_24h were highly correlated.

### Differentially expressed gene identification in leaves of under flooding stress

**Fig. 1:**
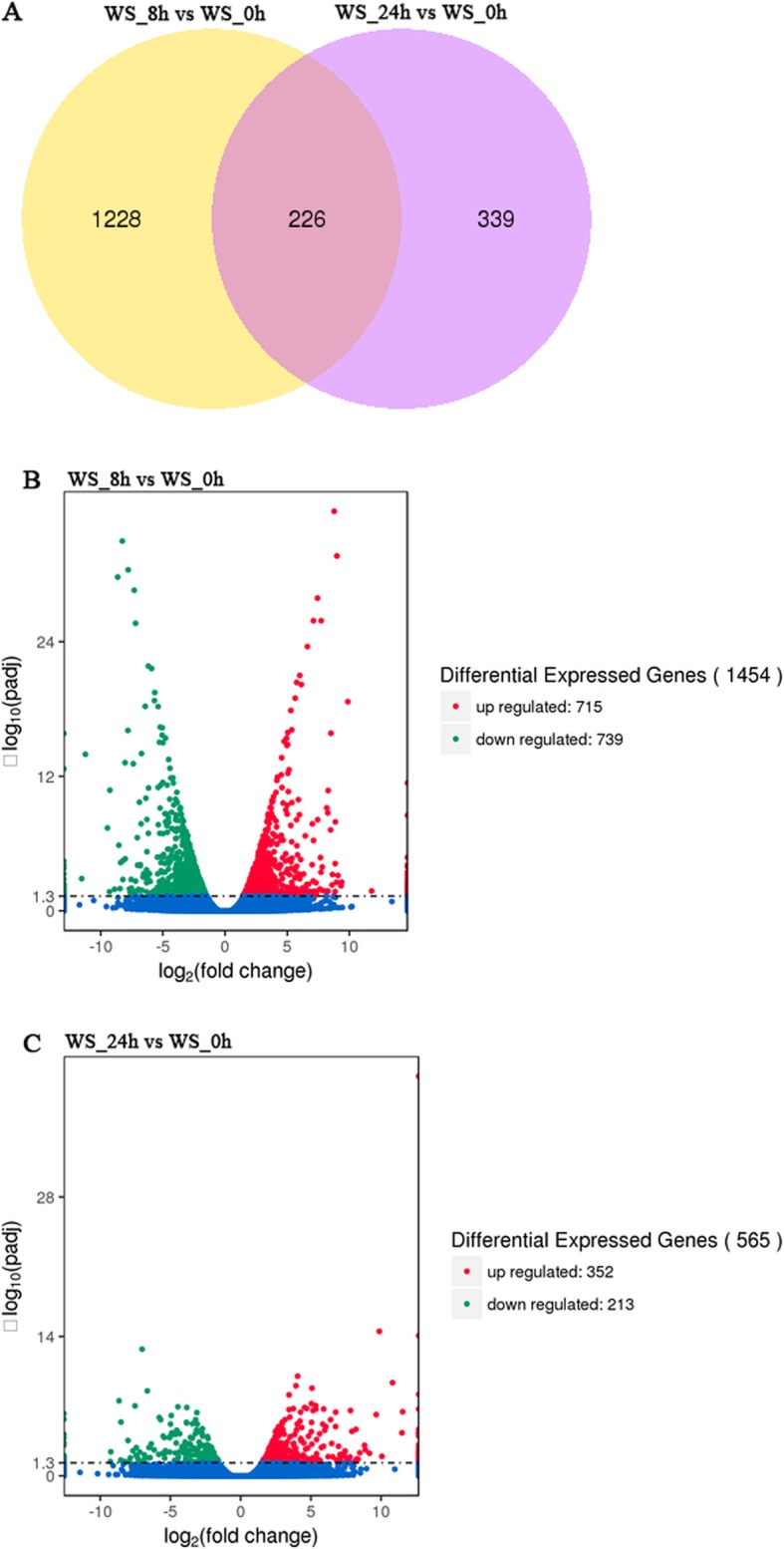
Venn diagrams and Volcano map of DEGs under flooding treatment in orchardgrass plantlets. **a**: Comparison of differentially expressed genes at 8 h and 24 h after flooding treatment with treatment for 0 h (CK); The numbers of DEGs with common were shown in the overlapping region. The total numbers of up- or down-regulated genes in each treatment were the sum of the numbers in each circle. **b**, **c**: Up-regulation and down-regulation of differential genes in 8 h, 24 h flooding treatment. The numbers of DEGs with common or opposite expression change tendencies between treatments were shown in the overlapping regions. The total numbers of up- or down-regulated genes in each treatment were the sum of the numbers in each circle

To screen the differentially expressed genes (DEGs), we used DESeq software for differential gene analysis, and q-value (p-adj) < 0.05 was defined as DEGs. Integration the reproducibility of the biological replicates, we found 2019 DEGs in leaves following the flooding stress (Table S2, S3). Among them, 226 DEGs were identified in response to both 8 h and 24 h flooding stress (Fig. 1a). A total of 1454 DEGs were found in response to 8 h flooding stress, of which 715 DEGs were up-regulated and the remaining 739 DEGs were down-regulated (Fig. 1b). A total of 565 DEGs were found responding to 24 h flooding stress, of which 352 DEGs were up-regulated and 213 DEGs were down-regulated (Fig. 1c). There were more DEGs found in the 8 h flooding stress than in the 24 h, suggesting that the response mechanism of orchardgrass to the early flooding stress period was more dramatic and complicated.

### GO enrichment analysis of the DEGs

To better understand the response mechanism of orchardgrass to flooding stress, we performed GO enrichment analysis on the biological functions of all DEGs. Correction *p*-value < 0.05 can be used as an enrichment project for a certain function. DEGs GO enrichment histogram visually reflected the distribution of DEGs in GO terms of biological processes, cellular components, and molecular function enrichment.

**Fig. 2:**
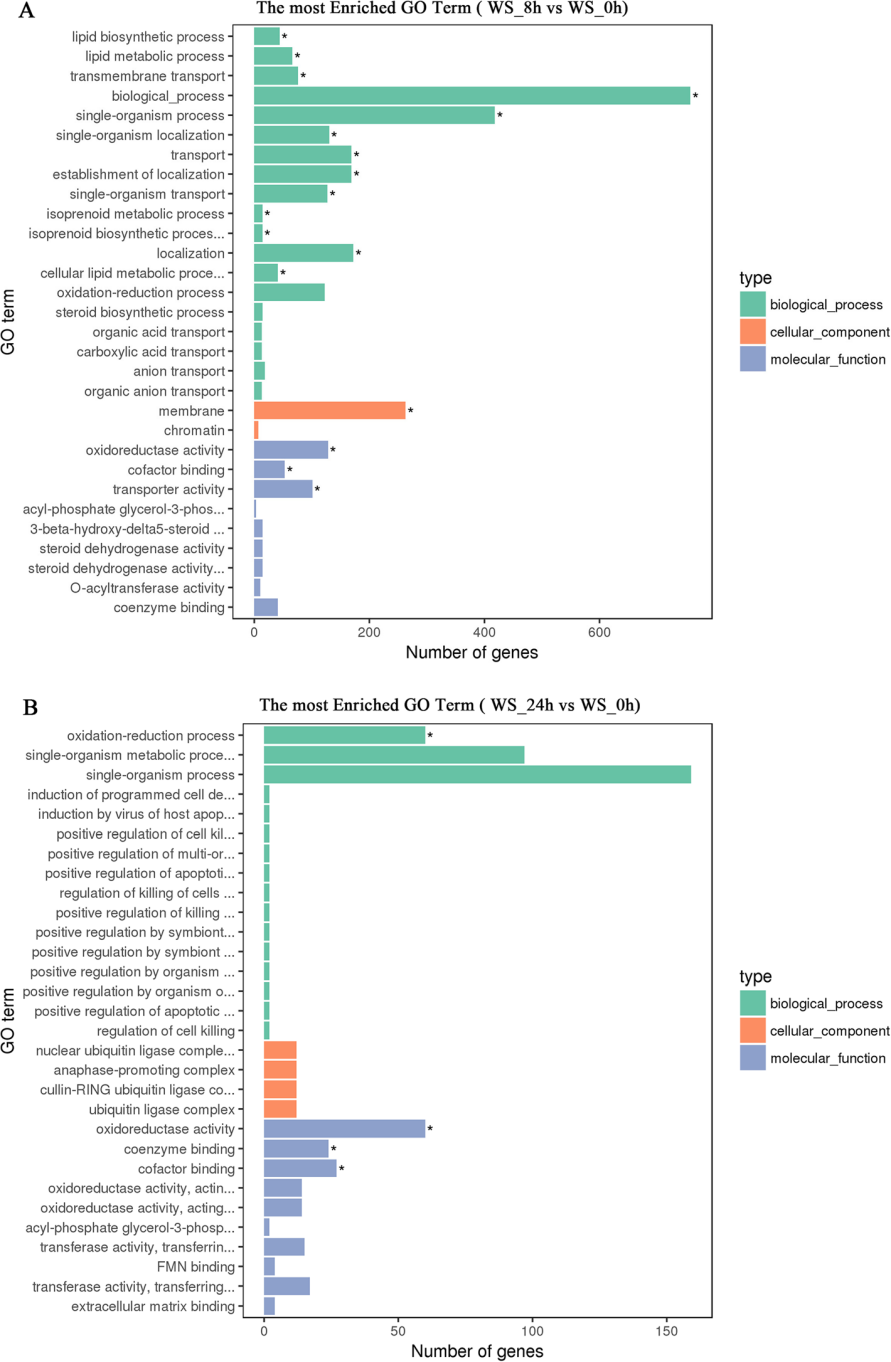
GO functional classification of the control and flooding stress orchardgrass seedling. “*” was an enriched GO term. **a**: comparison between 8 h of flooding stress and 0 h stress (CK); **b**: comparison between 24 h of flooding stress and 0 h stress (CK)

Comparing DEGs in 8 h flooding stress with those in 0 h (CK) treatment, DEGs enriched in biological processes were the most abundant and significantly enriched, mainly involving, single-organism process (GO:0044699), transport (GO:0006810) and establishment of localization (GO:0051234); followed by membrane (GO:0016020) in the category of cell components. The DEGs enriched in molecular function were the least, mainly involving oxidoreductase activity (GO:0016491) and transporter activity (GO:0005215) (Fig. 2a, Table S4). Also, we found that the number of DEGs was down-regulated more than that of up-regulation (Figure S3A). In the down-regulated most enriched GO term, the DEGs have multiple enrichment terms, among which the DEGs in the biological process were the most abundant (Figure S2A). While in the up-regulated most enriched GO term, there was no DEG enrichment. We speculated that the DEGs were mostly inhibited by 8 h of flooding stress, especially for those enriched in the biological process.

Comparing 24 h flooding stress with those in 0 h (CK) treatment, the DEGs enriched in the biological process mainly involved the oxidation-reduction process (GO:0055114), and the DEGs enriched in the molecular function were more than the biological processes, including oxidoreductase activity (GO:0016491), co-enzyme binding (GO:0050662), and co-factor binding (GO:0048037) (Fig. 2b, Table S5). We found that after 24 h of flooding stress, the up-regulated DEGs had significant enrichment in GO terms (Figure S2B), while the down-regulated DEGs had no enrichment. Besides, most of the DEGs enriched in biological processes, molecular functions, and cellular components were up-regulated (Figure S3B). Interestingly, this is exactly the opposite of the enrichment mode of DEGs treated with 8 h of flooding stress.

Through a comprehensive comparison of two flooding stress (8 h and 24 h) DEGs most enriched GO Term (Figure S2A, B), we found that oxidoreductase activity, co-factor binding, and oxidation-reduction process were highly present in the 8 h and 24 h of flooding stress. The DEGs enriched in the oxidation-reduction process and oxidoreductase activity were down-regulated by 8 h of flooding stress. Under 24 h of flooding treatment stress, the DEGs enriched in oxidoreductase activity and oxidation-reduction process were promoted and up-regulated. In summary, it suggested that the DEGs enriched in these two GO terms (oxidoreductase activity, oxidation-reduction process) played a crucial role in the response to flooding stress.

### KEGG pathways enrichment analysis of the DEGs

When plants encounter stress, different genes coordinate with each other to regulate biological functions. The significantly enriched pathway of DEGs can identify the most important biochemical metabolic pathways and signal transduction pathways in plant responses to stress. Here, we used KOBAS (2.0) for pathway enrichment analysis. FDR ≤ 0.05 indicated that the differential gene was significantly enriched in a certain pathway.

**Fig. 3:**
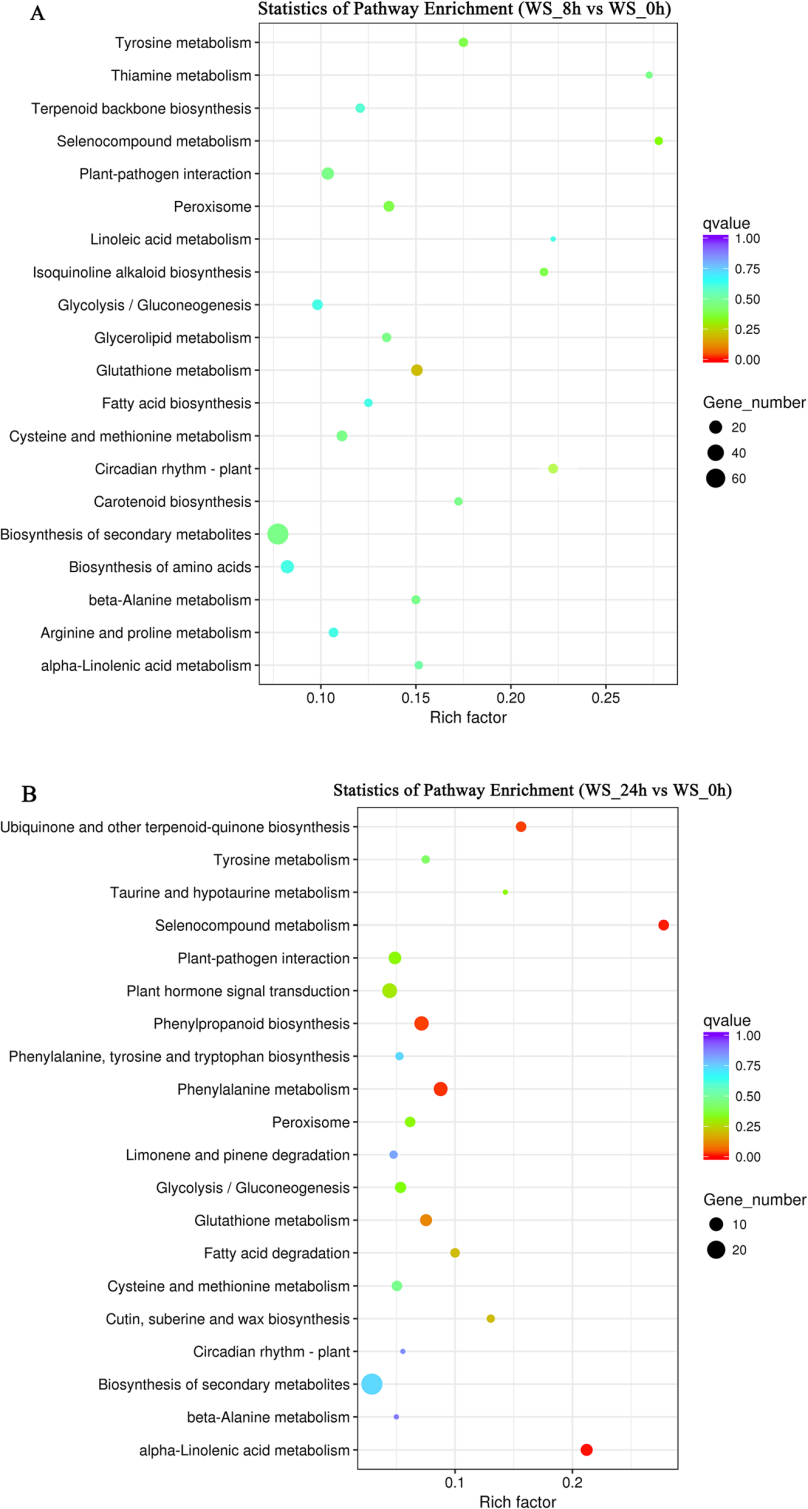
Differential gene KEGG enrichment scatter plot of the control and flooding stress orchardgrass seedling. The ordinate indicates the name of the path and the abscissa indicates the Rich factor. The size of the dot indicates how many DEGs were in the pathway, and the color of the dot corresponds to a different q-value range. **a**: comparison between 8 h of flooding stress and 0 h stress (CK); **b**: comparison between 24 h of flooding stress and 0 h stress (CK)

The key pathways of orchardgrass seedling in response to 8 h flooding stress were glutathione metabolism, selenocompound metabolism, peroxisome, tyrosine metabolism, isoquinoline alkaloid biosynthesis, biosynthesis of secondary metabolites, plant-pathogen interaction, thiamine metabolism, carotenoid biosynthesis, beta-alanine metabolism, glycerolipid metabolism (Fig. 3a, Table S6).

The key pathways in response to 24 h flooding stress were alpha-Linolenic acid metabolism, selenocompound metabolism, phenylalanine metabolism, ubiquinone and other terpenoid-quinone biosynthesis, phenylpropanoid biosynthesis, glutathione metabolism, fatty acid degradation, cutin suberine and wax biosynthesis, plant hormone signal transduction (Fig. 3b, Table S7).

The pathways of significant enrichment of DEGs under 8 h flooding stress were different from 24 h stress. Only glutathione metabolism and selenocompound metabolism were found in both (8 h and 24 h) flooding treatments. The DEGs enriched in the glutathione metabolism pathway after 8 h flooding stress was more abundant than that in the 24 h flooding, suggesting that glutathione metabolism more responded to 8 h of flooding stress than the 24 h.

### Cluster analysis of the DEGs

KEGG enrichment analysis was performed on all DEGs at 8 h and 24 h compared with 0 h respectively. We screened the significantly enriched 58 DEGs from nine pathways (glutathione metabolism, selenocompound metabolisms, peroxisome, tyrosine metabolism, isoquinoline alkaloid biosynthesis, alpha-Linolenic acid metabolism, phenylalanine metabolism, ubiquinone and other terpenoid-quinone biosynthesis, phenylpropanoid biosynthesis).

**Fig. 4:**
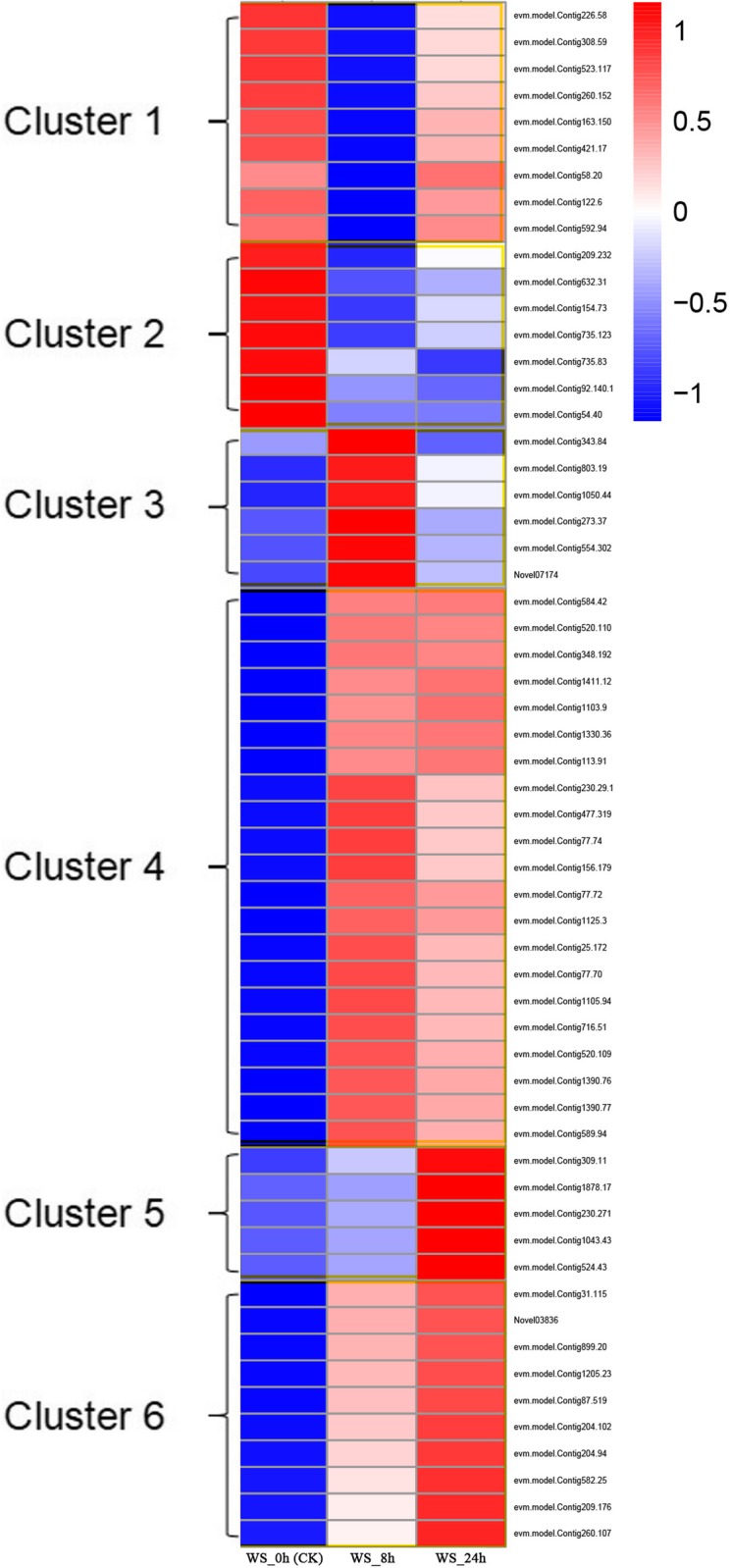
FPKM hierarchical clustering diagram of 58 DEGs in three-time points. The log_10_ (FPKM+ 1) values were scaled and clustered, with red indicating a highly expressed gene and blue indicating a low expressed gene. Colors from red to blue, indicating log_10_ (FPKM+ 1) from big to small. Cluster1, 2, 3, 4, 5, 6: Six patterns of 58 DEGs at different time points of flooding stress

The cluster heat map clearly showed that the expression patterns of 58 DEGs at different flooding time points were clustered into 6 clusters (Fig. 4). Among them, the expression patterns of DEGs clustered in Clusters1 and Clusters3 were the opposite. The main pathways involved in these two clusters are tyrosine metabolism and glutathione metabolism, as well as shared peroxisome. Compared with 0 h of flooding stress, the expression of DEGs was down-regulated, but both the 24 h stress DEGs and showed up-regulated patterns in Clusters1. This might indicate that under 8 h flooding stress, the expression of 9 DEGs in Cluster1 was inhibited. With the prolongation of flooding time, we hypothesized that the stressed plants could adjust themselves to cope with the stressful environment. Compared with the control group, the DEGs accumulated in Clusters2 were significantly down-regulated under flooding treatment, while the DEGs contained in Clusters4 and Clusters6 were significantly up-regulated. They were enriched in the pathways peroxisome, glutathione metabolism, selenocompound metabolism and phenylpropanoid biosynthesis, alpha-Linolenic acid metabolism, respectively (Table 2). In the Clusters5, Compared with 0 h (CK) flooding stress, the expression pattern of DEGs under 8 h was consistent with the control and down-regulated; while the expression pattern of DEGs was up-regulated at 24 h of flooding stress, contrary to the expression pattern of the control. In addition, we found that the DEGs contained in Cluster1, Cluster2, and Cluster3 were mainly derived from the 8 h treatment group, and the DEGs of Cluster4 were derived from 8 h and 24 h, while the DEGs of Cluster5 and Cluster6 were mainly derived from the enrichment pathway of the 24 h treatment group. This suggests that plants have a biological adaptation process in response to flooding stress to alleviate the damage caused by adversity. The flooding stress response of plants could be divided into three different stages, namely the alarm period, the acclimation period and the resistance period [27]. Studies have shown that the first stage of plant response to flooding stress is rapid induction of signals, the second stage is the adaptation stage activated by the first stage, and the third stage is the formation of aeration tissue at the root [28] .

**Table 2:** Expression patterns of 58 DEGs under different flooding stress

Cluster	Number of DEGs	DEGs expression mode			The main enriched pathway of DEGs
		WS_0h	WS_8h	WS_24h	
1	9	**↑**	**↓**	**↑**	Tyrosine metabolism, Peroxisome,
2	7	**↑**	**↓**	**↓**	Peroxisome
3	6	**↓**	**↑**	**↓**	Glutathione metabolism, Peroxisome
4	21	**↓**	**↑**	**↑**	Glutathione metabolism, Selenocompound metabolism
5	5	**↓**	**↓**	**↑**	alpha-Linolenic acid metabolism
6	10	**↓**	**↑**	**↑**	Phenylpropanoid biosynthesis, alpha-Linolenic acid metabolism

Note: “**↑**” means that the expression of DEGs is up-regulated, and “**↓**” means that the expression of DEGs is down-regulated

### Peroxidase is involved in the regulation of plant clearance of toxic ROS

**Fig. 5:**
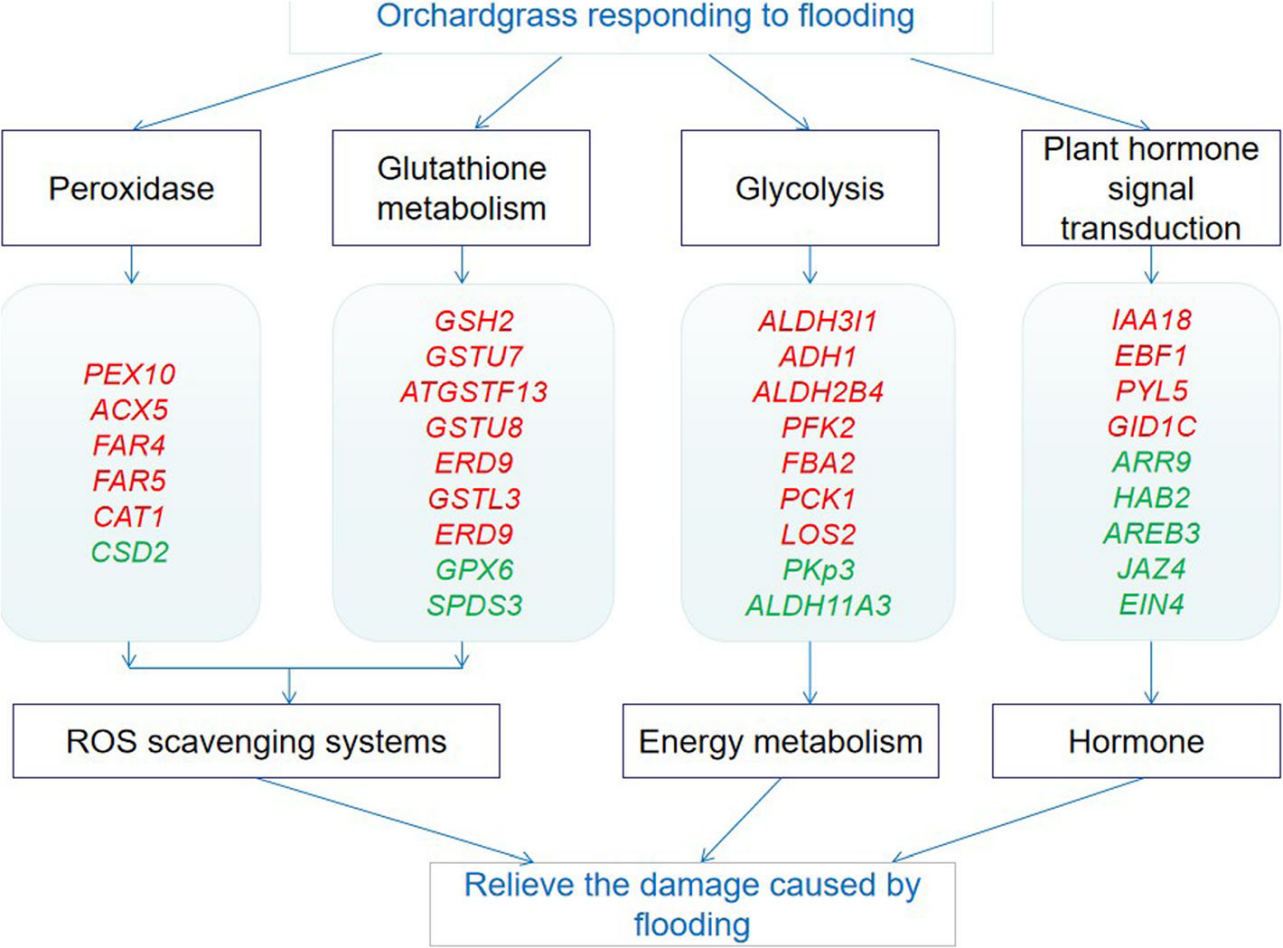
“Peroxidase”, “glutathione metabolism”, “glycolysis” and “phytohormone signal transduction” in response to flooding stress. Red means that the expression of DEGs is up-regulated, and green means that the expression of DEGs is down-regulated

Peroxisomes are important organelles equipped with a highly efficient reactive oxygen species (ROS) detoxification system, which is involved in many key metabolic processes such as oxidation of fatty acids, biosynthesis of ether lipids and detoxification of free radicals [29] (Fig. 5). It can enhance the adaptability of plants to stressful environments and plays a key role in plant redox signaling and lipid homeostasis.

Eleven DEGs were significantly expressed in the peroxisome pathway, of which five were up-regulated and eight down-regulated at 8 h of flooding stress (Table S9). The DEGs, *PEX10* were significantly up-regulated, indicating that it was promoted during peroxisome biogenesis. In abiotic stress, the total amount of intracellular peroxisomes could increase the total number of peroxisomes in addition to up-regulating ROS scavenging enzymes [30]. This increase was accompanied by the up-regulation of the peroxisome biosynthetic gene in *Arabidopsis*, including *PEX10* [31], indicating that *PEX10* was promoted during peroxisome biogenesis. Studies have shown that peroxidases proliferate in plants under stress conditions [32].

In recent years it has become apparent that peroxisomes play important roles in reactive oxygen metabolism [33]. Most reactive oxygen species metabolism is carried out in peroxisomes [34]. Excessive concentrations of ROS can cause oxidative damage to the peroxisome components exposed to it, but this can be eliminated by various ROS scavengers, such as non-enzymatic antioxidants: glutathione (GSH) and multiple antibodies. Oxidase: catalase (CAT), GSH reductase, ascorbate peroxidases (APX), peroxidase and superoxide dismutase (SOD) [35].

The differential gene-regulating *MPV17* was significantly down-regulated during ROS metabolism, indicating that ROS metabolism was inhibited under 8 h of flooding stress (Table S9). This might be caused by water shortage in the flooded environment and oxygen stress. Under oxidative stress conditions, the production of peroxisome ROS was enhanced and the clearance of ROS was insufficient [35], resulting in an imbalance of ROS metabolism. The differential gene that regulated *FAR5* was significantly up-regulated and promoted by stress. Differential genes encoding *CAT1* and *CSD2* were significantly up-regulated and down-regulated, respectively (Table S9). CAT was an important target for H_2_O_2_ scavengers. It is rich in plant peroxisomes while it has low substrate affinity. Regulating its catalytic activity might be the starting point to overcome this shortcoming, thereby making plant peroxisomes more effective in detoxification [36].

However, expressions of only five genes changed in the peroxidase pathway. Of them, four were up-regulated and one was down-regulated under 24 h of flooding stress (Table S9). *PXN* was a family of mitochondrial substrate carriers whose expression was promoted by flooding stress, while hypoxia was associated with decreased mitochondrial respiration [37]. Mitochondrial metabolism plays a key role in controlling the redox balance of whole plant cells [38]. The differential genes regulating *ACX5* and *FAR5* were significantly up-regulated, indicating that they responded positively to flooding stress to promote plant adaptation to stress.

### Glutathione metabolism is involved in the response of plants to flooding stress

Glutathione is an important non-enzymatic antioxidant when plants encounter abiotic stress. It can protect the sulfhydryl and membrane systems of structural proteins in cells, effectively remove excessive ROS components produced by plants during stress, and reduce peroxidative damage [39]. In response to 8 h of flooding stress, fourteen differential genes were significantly expressed during glutathione metabolism (Table S9), in which two genes regulating *GPX6* and *SPDS3* were down-regulated and twelve genes were significantly up-regulated including *GSTU7*, *ERD9*, *GSTU8*, *ATGSTF13*, *GSTL3* and *GSH2*. At 24 h of flooding stress, with a total of seven differential genes were associated with glutathione metabolism pathway (Table S9), including up-regulation of *ERD9*, *GSTU8*, *ATGSTF13*, *GSTL3* and down-regulation of *SPDS3* (Fig. 5). It has been reported that antioxidant genes such as *GST* and *GPX* are involved in flooding stress, suggesting that they activate the ROS elimination process [15].

### Glycolysis is involved in the stress of plants on flooding stress

Glycolysis is one of the important ways of energy production under hypoxic conditions. Maintaining the growth of glycolysis may be the key to plant survival. When plants are exposed to flooding, they are also accompanied by oxygen stress, which causes rapid changes of gene transcription, protein synthesis and degradation, and cellular metabolism [40]. Under hypoxic conditions, glycolysis is mainly through the fermentation pathway (rather than aerobic respiration), which is necessary for cell survival to generate energy and to recover carbon for other pathways [38]. *ADH* accelerates ethanol fermentation and allows glycolysis to provide ATP to plants during flooding, thereby improving the ability of plant adaptation to stress [41].

There were ten differential genes involved in glycolysis, of which two genes were significantly up-regulated and nine genes were significantly down-regulated in to response 8 h of flooding stress (Table S9). The up-regulated functional genes were *ALDH3I1* and galactose mutarotase-like superfamily protein. The down-regulated genes included galactose mutarotase-like superfamily protein, *PFK2*, *FBA2*, *ALDH11A3*, *LOS2*, *PCK1*, *PKp3*, and pyruvate kinase family protein. In response to 24 h of flooding stress, four DEGs were significantly up-regulated, and two DEGs were down-regulated (Table S9). Up-regulated genes were actate/malate dehydrogenase family protein, *ADH1*, and *ALDH2B4*. Galactose mutarotase-like superfamily protein and *PFK2* were regulated by two DEGs, respectively, and they were down-regulated (Fig. 5).

### Plant hormone-mediated pathways are involved in response to flooding stress

Hormones are important regulators of plant adaptation to environmental changes. Plant hormone signaling plays an important role in the stress resistance of many plants. In response to 8 h of flooding stress (Table S9), *SAUR*-like auxin-responsive protein family involved in the auxin-mediated tryptophan pathway was regulated by two differential genes, one of which was up-regulated and one down-regulated. In response to 24 h of flooding stress (Table S9), the differentially expressed genes regulating *GID1C* were significantly up-regulated during gibberellin-mediated diterpenoid biosynthesis. In the process of abscisic acid-mediated carotenoid biosynthesis, the four DEGs regulating *PYL* were up-regulated significantly, and one gene regulating *HAB2* was down-regulated significantly. In the ethylene-mediated metabolism of Hemi-amino acid and methionine, a DEG that regulates *EIN4* was up-regulated, and a DEG that regulates *EBF1* was up-regulated. The SUB1 locus contains three clusters of ethylene response factor (*ERF*) genes, in which the *SUB1A* allele *SUB1A-1* limits the growth of plants under flooding and confers flood tolerance [42].

### Validation of the DEGs by qRT-PCR analysis

To validate the accuracy and reproducibility of the RNA-seq results, we randomly screened ten DEGs for qRT-PCR, which were up- and down-regulated. The relative expression of each gene was calculated using the 2^-ΔΔCt^ method. The results showed that ten DEGs were differentially expressed between the control group (flooding for 0 h) and the stress-treated group (flooding for 8 h and 24 h) (Figure S4). The qRT-PCR verification results were highly correlated with the RNA-seq data (Figure S5), confirming the reproduci bility of the RNA-Seq data.

## Discussion

In this study, high-throughput RNA sequencing technology was used to compare the expression profiles of DEGs in the leaves of orchardgrass cv. Dianbei in 8 h and 24 h under flooding stress. This study provided insights into a better understanding of the molecular mechanisms of flooding tolerance in orchardgrass.

By comparing the bioinformatics analysis of the obtained transcript data of orchardgrass, we found that many biological processes, cell components and molecular processes in the leaves of orchardgrass were affected by flooding stress. In response to stress, oxidoreductase activity in the GO enrichment term was significantly enriched at both time points, and it was also significantly up-regulated in the GO enrichment entry in cucumber exposed to flooding stress [16]. At 8 h and 24 h of stress, the differential genes were significantly expressed in the two GO terms entries of oxidation-reduction process and oxidoreductase activity, indicating that different groups of oxidoreductase genes were involved in maintaining redox balance or alleviating sputum-induced oxidative stress [1] suggesting that oxidoreductase activity played an important role in plant response to flooding stress. In the study, in the GO enriched molecular function category, binding, catalytic activity and transporter activity were highly present, which was consistent with the analysis of the short-term smear stress on the rhizome transcriptome of *Taxodium* [19].

In this experiment, a total of 102 pathways were found in the list of KEGG differential gene enrichment pathways in the 8 h of flooding treatment, and 77 pathways in the 24 h of flooding treatment. The pathways on the top list were glutathione metabolism, peroxisome, selenium compound metabolism, tyrosine metabolism, biosynthesis of secondary metabolites, plant-pathogen interaction, plant hormone signal transduction, suggesting that flooding impacted a wide range of physiological processes. We have found that these processes have been reported in studies of other plant flooding stresses. Flooding stress should be considered as a compound stress consisting of several potential changes in the internal and external sources of ethylene, carbon dioxide, O_2_, ROS and plant toxins [43].

ROS is a by-product of basic cellular processes such as plant photosynthesis and respiration, and is produced by the joint action of several enzymes [44]. One of the main sources of active oxygen in plants is a reaction mediated by coagulin oxidase, which is responsible for the conversion of O_2_^−^ to superoxide anion, resulting in the production of hydrogen peroxide [45]. ROS are important signals in plants and key regulators of a variety of processes including metabolism, growth and development, response to abiotic and biotic stresses, solute transport, autophagy and programmed cell death [46].

Accumulation of ROS is an important indicator of abiotic stress at the molecular level. Once the ROS removal system is disrupted, the plant will suffer from oxidative stress. In general, plants can detoxify ROS by producing different types of antioxidants. Antioxidants are divided into enzymatic and non-enzymatic antioxidants. Enzyme antioxidants include superoxide dismutase (SOD), peroxidase (POD), catalase (CAT) and glutathione reductase (GR), and ascorbic acid, glutathione, tocopherol, and carotenoids are non-enzymatic antioxidants [47]. Many antioxidant enzymes are the key to the survival of many plants under varying degrees of flooding stresses [19]. In the study, down-regulation of *CSD2* encoding SOD and up-regulation of ROS scavenging genes (*CAT1* and *glutathione S-transferase*) were observed under flooding stress. SOD is the first line of defense for removing active oxygen in plants [48]. At the core of the enzyme protection system, the flood tolerance of plants is directly related to the strength of SOD activity. Its main function is to catalyze the disproportionation reaction of superoxide anion into hydrogen peroxide, which is further decomposed to water and oxygen by conversion of POD, CAT, GR and APX [49]. Down-regulation of *CSD2* might increase ROS levels in the leaves of flooded orchardgrass seedlings. Catalase may be involved in the control of hydrogen peroxide (H_2_O_2_) levels by converting H_2_O_2_ to O_2_.This may be the reason why Dianbei is resistant to flooding. Similar to this study, the ROS scavenging genes (encoding SOD, CAT and *glutathione-S-transferase*) were significantly expressed in rape. The difference is that the gene encoding CAT is down-regulated and the gene encoding SOD is down-regulated [50]. Similar findings were found in cucumber [45], *Maize* [2], and *A. philoxeroides* [51] flooding studies.

Glycolysis is one of the important ways of energy production under hypoxic conditions. Maintaining the growth of glycolysis may be the key to plant survival [20]. The glycolysis and fermentation processes are enhanced under flooding conditions, including ethanol fermentation (PDC and ADH catalysis) and lactic acid fermentation (LDH catalysis). ADH accelerates ethanol fermentation and allows glycolysis to provide ATP to plants during flooding, thereby improving the ability of plants to adapt to adversity [41]. Under flooding conditions, plants regulate mitochondrial respiration to glycolysis by fermentation of pyruvate decarboxylase and alcohol dehydrogenase [52]. In this study, up-regulated expression of *ADH* verified that ADH plays an important role in plant response to flooding. Comparative transcriptome analysis of other plants has also identified glycolysis and upregulation of fermentation genes as common responses to flooding, such as Jatropha [53], *Sesbania cannabina* [54], soybean [55]. Our results supported the longstanding notion that flooding promotes anaerobic respiration, as observed by the up-regulation of DEGs encoding enzymes involved in glycolysis and fermentation.

Plant hormones play an important role in plant resistance to stress. Plant growth regulators are induced to participate in a signal cascade that affects cellular responses under flooding stress, including reduction of ethylene (C_2_H_4_), abscisic acid (ABA), gibberellin (GA), auxin (IAA), and cytokinin (CK). Many genes involved in hormone production and signaling were found to be affected in both roots and shoots of flooded plants [40]. Ethylene has long been known to be involved in responses to hypoxia and is thought to contribute to adventitious root production and aerenchyma formation [56]. The Ethylene response factor (*ERF*) represented the highest number of significantly expressed TFs under hypoxic conditions. In this study, the expression of *EBF1/2* in orchardgrass leaves was significantly increased under flooding conditions, suggesting that flooding is related to ethylene synthesis and perception. This proves that ethylene plays an important role in plant flooding stress. The phytohormone ethylene mediates physiological, developmental, and stress responses by activating ethylene-responsive factors (*ERFs*) belonging to the multi-gene family of transcription factors [57]. In Arabidopsis, *ERF1* plays a positive role in salt, drought, and heat stress tolerance by regulating stress-specific gene, and by integrating jasmonic acid, ethylene, and abscisic acid signals [58]. This has also appeared in other studies of plant flooding stress. These results indicate that plants need to induce genes associated with hypoxia response through *ERF* regulation, but further studies are needed to test other *ERFs*. ABA has long been recognized as a key factor in abiotic stress response in plants [59]. The major ABA receptor (*PYR / PYL / RCAR*) responsible for stomatal closure is a soluble protein [60]. In this study, *PYL2*, *PYL5*, and *RCAR3* were significantly expressed in the abscisic acid-mediated carotenoid biosynthesis. This also suggests that ABA plays an important role in responding to flooding stress. IAA is the main biologically active auxin in plants [61]. In the present study, the gene IAA18 encoding indole-3-acetic acid was up-regulated. The abundance of IAA activates the *MAPK* signal cascade at intervals, leading to the formation of adventitious roots, which is very important for plants in flooded environments [62]. The formation of adventitious roots is an important morphological change in plants in response to flooding or hypoxia.

By qRT-PCR analysis, it was found that the expression patterns of genes encoding GSTL3, ATGSTF13, CaMCML, GSTU7, GSTU8, CPNIFS, HMGCL, MPV17, and DND1 were significantly expressed under flooding stress, indicating that they play an important role in flood resistance of orchardgrass. Among them, we found that compared to the control group, genes encoding GST (GSTL3, ATGSTF13, GSTU7, GSTU8) were significantly induced under water flooding, indicating that these genes regulate GST in an important role in the ROS clearance system. Similar research conclusions have also appeared in the study of resistance to rape [17], cucumber [45] and tall fescue [63].

## Conclusion

This study is the first time that a transcriptome analysis of orchardgrass leaves in response to short-term flooding stress has identified a total of 2019 differentially expressed genes. Oxidoreductase activity, cofactor bling and oxidation -reduction process are highly present in GO-enriched terms, suggesting that the leaves of orchardgrass have a complex oxidoreductase process in response to flooding stress. This study also found that peroxidase, glutathione, glycolysis and phytohormone signal transduction play important roles in the domestication of orchardgrass. This study laid a foundation on a further study of genetic control of flooding tolerance for orchardgrass.

## Materials and methods

### Plant materials and stress conditions

The test material is “Dianbei”, which is an orchardgrass (*Dactylis glomerata* L.) variety with strong flood tolerance.

Orchardgrass seeds of uniform size and fullness were screened and sown in pots containing sand and soil in a ratio of 1: 2 (V: V) (diameter 10 cm, height 8 cm) (Chongqing, China). After sowing, the pots were placed in a light incubator. The relative humidity in the light incubator is constant, the temperature is set to 25 °C/20 °C (day / night), the photoperiod is 14 h / 10 h (day / night), and the photon flux density is about 300 μmol m ^− 2^ s ^− 1^. Huguelan solution was supplied regularly during shoot growth. Huguelan solution was supplied regularly during the seedling period.

Three months after sowing, select well-grown orchard grasses for testing. The pot was placed in a water tank (length 80 cm; width 57 cm; height 50 cm) filled with room temperature water, and the water level was kept higher than the highest part of the plant as the treatment group, and the control group was a normal-growing orchardgrass plant. The 24 h samples were first flooded, and after 16 h, the 8 h samples were flooded, with three biological replicates per treatment. When all treatments were completed, the leaves of the orchard grass from three treatments (0 h, 8 h, and 24 h) were cut at the same time, put into sterile tubes, quickly frozen with liquid nitrogen, and stored at − 80 °C.

### RNA extraction and library construction

Total RNA was extracted from the samples using an RNA prep pure plant kit (Tiangen Biochemical Technology Company, Beijing, China). (1) Analysis of the degree of RNA degradation and contamination by running agarose gel electrophoresis; (2) Nanodrop testing (OD260/280 ratio); (3) qubit accurately quantification of RNA concentration; (4) Agilent 2100 detecting RNA integrity. Finally, only RNA samples that passed the quality test were selected for RNA-Seq analysis. Transcriptome sequencing was conducted on an Illumina Hiseq 4000 (Illumina, San Diego, CA, USA) at the Novogene Bioinformatics Institute (Beijing, China).

Approximately 3 μg of RNA for each sample was used for library construction, using the NEBNext® Ultra™ Directional RNA Library Prep Kit for Illumina® (NEB, USA). Transcriptome sequencing was conducted on an Illumina Hiseq 4000 (Illumina, San Diego, CA, USA) at the Novogene Bioinformatics Institute (Beijing, China). The orchardgrass genome reference was used for read mapping (http://orchardgrassgenome.sicau.edu.cn/). All of the sequencing data was deposited into the NCBI SRA database.

### Reads mapping

Filtering the original sequencing sequence obtained by sequencing: 1) removing the reads with adapters;(2) removing N (N means that the base information cannot be determined) the ratio of reads greater than 10%;(3) Remove low-quality reads (Q_phred_ < = 20 bases account for more than 50% of the entire read length of reads). We selected HISAT [64] software to perform genomic localization analysis on the filtered sequence. In the analysis process, the software default parameters were used. Reference to the genome and gene model of orchardgrass [65]. The HISAT algorithm is mainly divided into three parts: (1) the entire sequence of the sequencing sequence was aligned to the single exon of the genome; (2) the sequencing sequence was segmentally aligned to the two exons of the genome; (3) the sequencing sequences into more than three (including three) exons in the genome.

In RNA-seq analysis, we can estimate gene expression levels by counting the number of sequencing reads that are located in the genomic region or exon region. In this experiment, each sample was analyzed for gene expression level using HTSeq [66] software, and the model used was union. To make the estimated gene expression levels of different genes and different experiments comparable, the concept of FPKM was introduced. FPKM is currently the most commonly used method for estimating gene expression levels. The input data of gene differential expression analysis was the readcount data obtained from the gene expression level analysis. The analysis was mainly divided into three parts: 1) first normalized the readcount; 2) then calculated the hypothesis test probability (*p*-value) according to the model 3) Finally, multiple hypothesis test corrections were performed to obtain the FDR value (error discovery rate). Analysis of gene differential expression using DESeq [67] software.

### GO and KEGG enrichment analysis of differentially expressed genes

The software method used in our analysis of GO enrichment analysis was GOseq, which is based on the Wallenius non-central hyper-geometric distribution. Compared to the ordinary hyper-geometric distribution, this distribution is characterized by the fact that the probability of extracting an individual from a certain category is different from extracting an individual from a outside certain category and the probability is different. It is estimated by the length of the gene, so that the probability of GO term being enriched by differential genes can be calculated more accurately. Pathway Significant Enrichment Analysis Using Pathway in the KEGG database, hypergeometric tests were used to find Pathway that was significantly enriched in differentially expressed genes compared to the entire genome background.

### Validation of RNA-Seq data by qRT-PCR

Ten genes were randomly selected from the DEGs to verify differential gene expression levels with three biological repeats. Gene-specific qRT-PCR primers were designed using Primer3 software (http://primer3.ut.ee/), for ten selected genes with the sequence data in 3’UTR (Table S8). PrimeScript RT reagent Kit with gDNA Eraser (Takara, Chongqing, China) is a dedicated reverse transcription kit for RT-PCR reactions, following the manufacturer’s protocol. qPCR was performed using TB Green™ Premix Ex Taq™ II (Takara) on Applied Biosystems 7500 Fast Real-Time PCR System. The volume of the qRT-PCR reaction was 10 μl, containing 1 μl cDNA, 5 μl TB Green Premix Ex Taq II (Tli RNaseH Plus) (2×), 0.4 μl ROX Reference Dye II (50×), 0.8 μl of the forward and reverse primers, and 7 μl ddH_2_O. Actin was used as the endogenous reference gene. The reaction had three phases. Stage 1 (hold): 95 °C 30 s 1 Cycle, Stage 2 (PCR reaction): 95 °C 5 s,60 °C 20 s,40 Cycles, Stage 3 (melting curve analysis): 95 °C 15 s, 60 °C 1 h,95 °C 15 s. All PCR reactions were normalized using the Ct value corresponding to the endogenous reference gene (translationally controlled tumor protein [*TCTP*] [68]). Three biological replicates were generated and three measurements were performed on each replicate.

### Statistical analysis

Data analysis and mapping were performed using IMS SPSS Statistics and OriginPro8.5, respectively. All values are expressed as mean ± SD. All the values were expressed as mean ± SD. Comparison between control and flooding groups was estimated with One-way ANOVA followed by LSD post hoc test, and *P*_-value_ < 0.05 was considered to be statistically significant.

## Supplementary information

**Additional file 1: Table S1.** Summary of RNA-seq sequence a nalysis of orchardgrass. **Table S2.** Differential analysis results of all DEGs suffering flooding at WS_8h vs WS_0h in leaves. **Table S3.** Differential analysis results of all DEGs suffering flooding at WS_24h vs WS_0h in leaves. **Table S4.** GO enrichment analysis of all DEGs suffering flooding at WS_8h vs WS_0h in leaves. **Table S5.** GO enrichment analysis of all DEGs suffering flooding at WS_24h vs WS_0h in leaves. **Table S6.** KEGG Pathway enrichment analysis of all DEGs suffering flooding at WS_8h vs WS_0h in leaves. **Table S7.** KEGG Pathway enrichment analysis of all DEGs suffering flooding at WS_24h vs WS_0h in leaves. **Table S8.** The primers used in the qRT-PCR analysis.

**Additional file 2: Table S9.** Analysis of 4 key pathways in response to flooding stress.

**Additional file 3: Figure S1.** Correlation analysis under different time flooding stress. **Figure S2.** GO enrichment analysis of down-regulated DEGs suffering flooding at WS_8h vs WS_0h and up-regulated DEGs suffering flooding at WS_24h vs WS_0h in leaves. **Figure S3.** GO functional classification of all DEGs suffering flooding at WS_8h vs WS_0h and WS_24h vs WS_0h in leaves. **Figure S4.** qRT-PCR verification results of ten DGEs in control and treatment groups. **Figure S5. **Correlation between qRT-PCR and RNA sequencing for the ten selected genes.

## Data Availability

All data produced by the study are disclosed in the manuscript and the additional files. The raw data of this study was stored in the NCBI (National Coalition Building Institute) SAR (Sequence Read Archive) database, which can be viewed in BioProject (ID: PRJNA554779).

## Abbreviations

DEGs: Differentially expressed genes
ROS: Reactive oxygen species
*PEX10*: *Peroxin 10*
*MPV17*: *Peroxisome membrane 22 KDA*
*FAR5*: *Fatty acid reductase 5*
*CAT1*: *Catalase 1*
*CSD2*: *Copper/zinc superoxide dismutase 2*
*PXN*: *Mitochondrial substrate carrier*
*ACX5*: *Acyl-CoA oxidase 5*
*GPX6*: *Glutathione peroxidase 6)*
*SPDS3*: *Spermidine synthase 3*
*GSTU7*: *Glutathione S-transferase tau 7*
*ERD9*: *Glutathione S-transferase family protein*
*GSTU8*: *Glutathione S-transferase TAU 8*
*ATGSTF13*: *Glutathione S-transferase family protein*
*GSTL3*: *Glutathione S-transferase family protein*
*GSH2*: *Glutathione synthetase 2*
*ADH*: *Alcohol dehydrogenase*
*ALDH3I1*: *aldehyde dehydrogenase 3I1*
*PFK2*: *Phosphfructokinase 2*
*FBA2*: *Fructose-bisphosphate aldolase 2*
*ALDH11A3*: *Aldehyde dehydrogenase 11A3*
*LOS2*: *Enolase2*
*PCK1*: *Phosphoenolpyruvate carboxykinase 1*
*PKp3*: *Plastidial pyruvate Kinase 3*
*ALDH2B4*: *Aldehyde dehydrogenase 2B4*
*GID1C*: *Alpha/beta-Hydrolases superfamily protein*
*PYL*: *Polyketide cyclase/dehydrase and lipid transport superfamily protein*
*HAB2*: *Homology to ABI2*
*EIN4*: *Signal transduction histidine kinase, hybrid-type, ethylene sensor*
*EBF1*: *EIN3-binding F box protein 1*
qRT-PCR: quantitative real-time PCR
