# Supplementary material for: Transcriptome analysis on responses of orchardgrass (Dactylis glomerata L.) leaves to a short term flooding
Source: Hereditas. 2020 May 17;157:20. doi: 10.1186/s41065-020-00134-0 (PMC7232843; doi:10.1186/s41065-020-00134-0)
Supplement: Supplementary file 3 — Additional file 3: Figure S1. Correlation analysis under different time flooding stress. Figure S2. GO enrichment analysis of down-regulated DEGs suffering flooding at WS_8h vs WS_0h and up-regulated DEGs suffering flooding at WS_24h vs WS_0h in leaves. Figure S3. GO functional classification of all DEGs suffering flooding at WS_8h vs WS_0h and WS_24h vs WS_0h in leaves. Figure S4. qRT-PCR verification results of ten DGEs in control and treatment groups. Figure S5. Correlation between qRT-PCR and RNA sequencing for the ten selected genes. [file 41065_2020_134_MOESM3_ESM.ppt]

## Slide 1
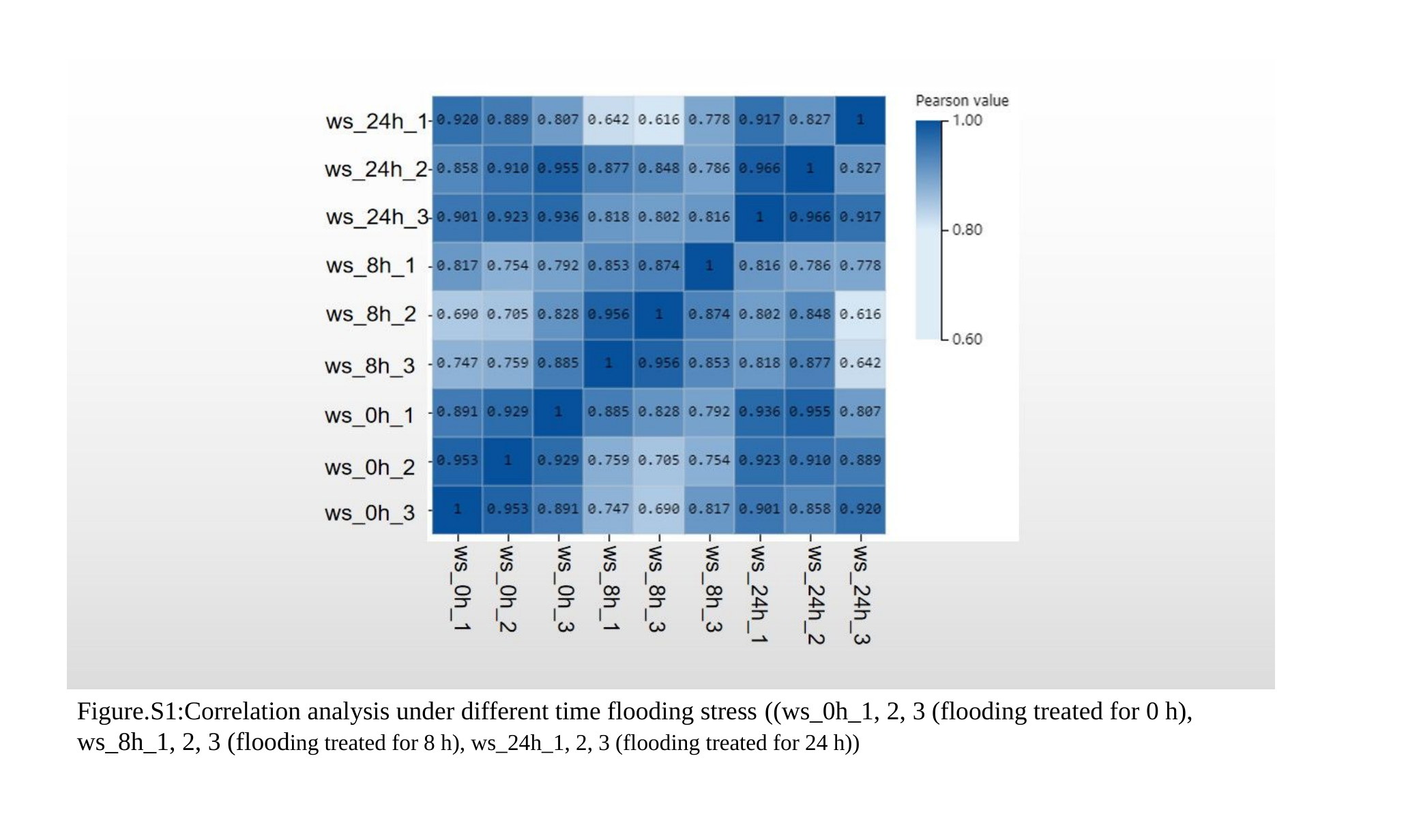

Figure.S1:Correlation analysis under different time flooding stress ((ws_0h_1, 2, 3 (flooding treated for 0 h), ws_8h_1, 2, 3 (flooding treated for 8 h), ws_24h_1, 2, 3 (flooding treated for 24 h))

## Slide 2
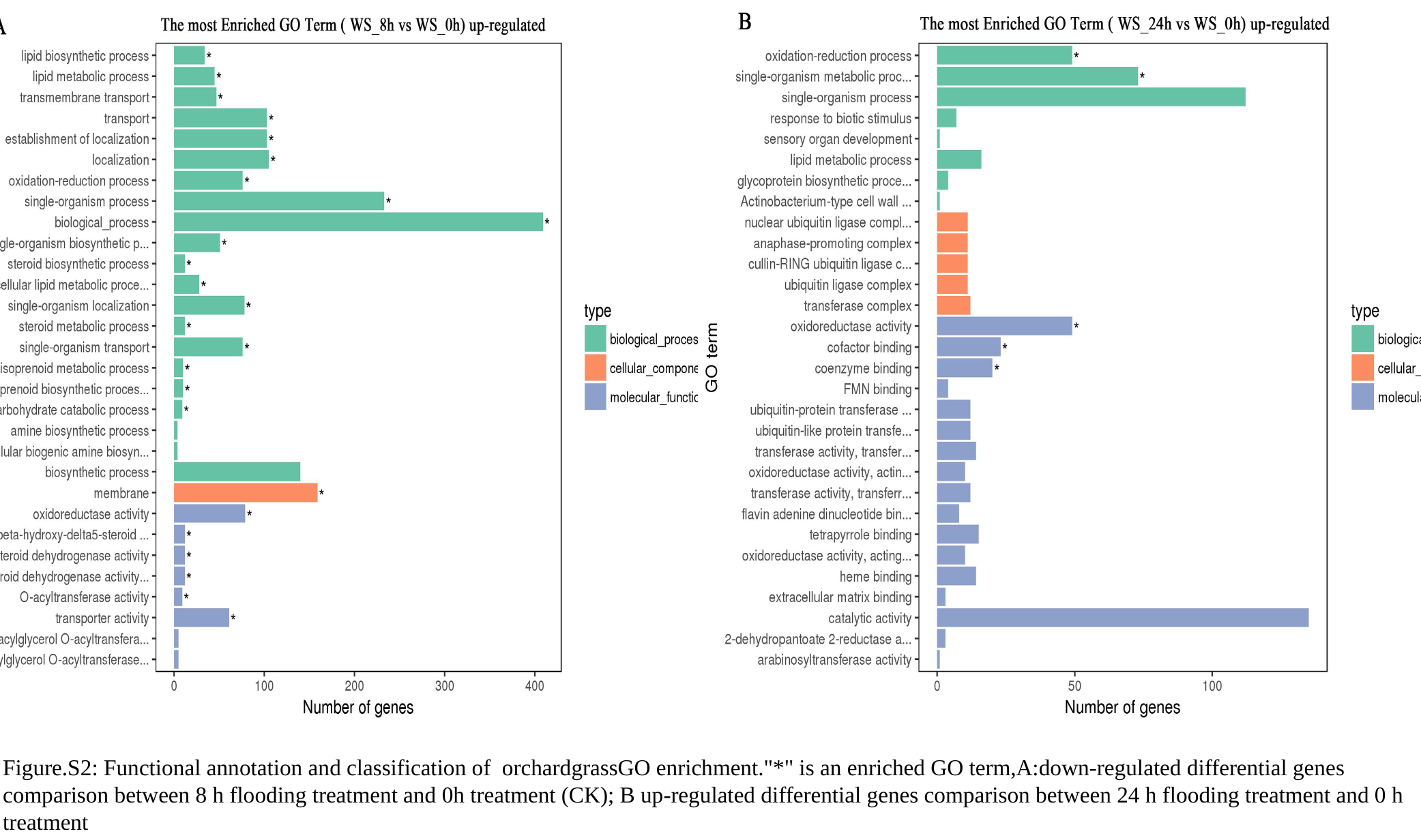

Figure.S2: Functional annotation and classification of orchardgrassGO enrichment."*" is an enriched GO term,A:down-regulated differential genes comparison between 8 h flooding treatment and 0h treatment (CK); B up-regulated differential genes comparison between 24 h flooding treatment and 0 h treatment

## Slide 3
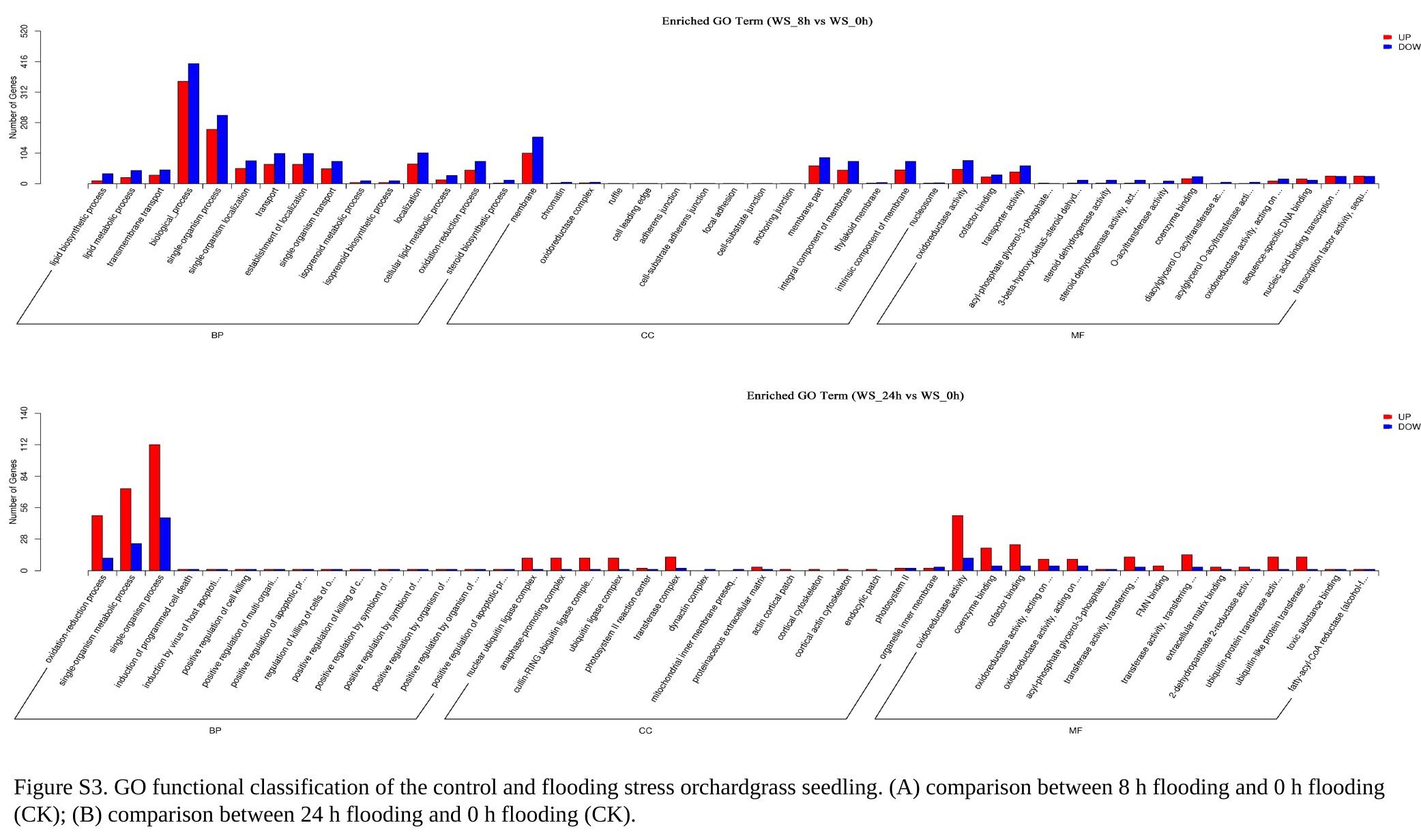

Figure S3. GO functional classification of the control and flooding stress orchardgrass seedling. (A) comparison between 8 h flooding and 0 h flooding (CK); (B) comparison between 24 h flooding and 0 h flooding (CK).

## Slide 4
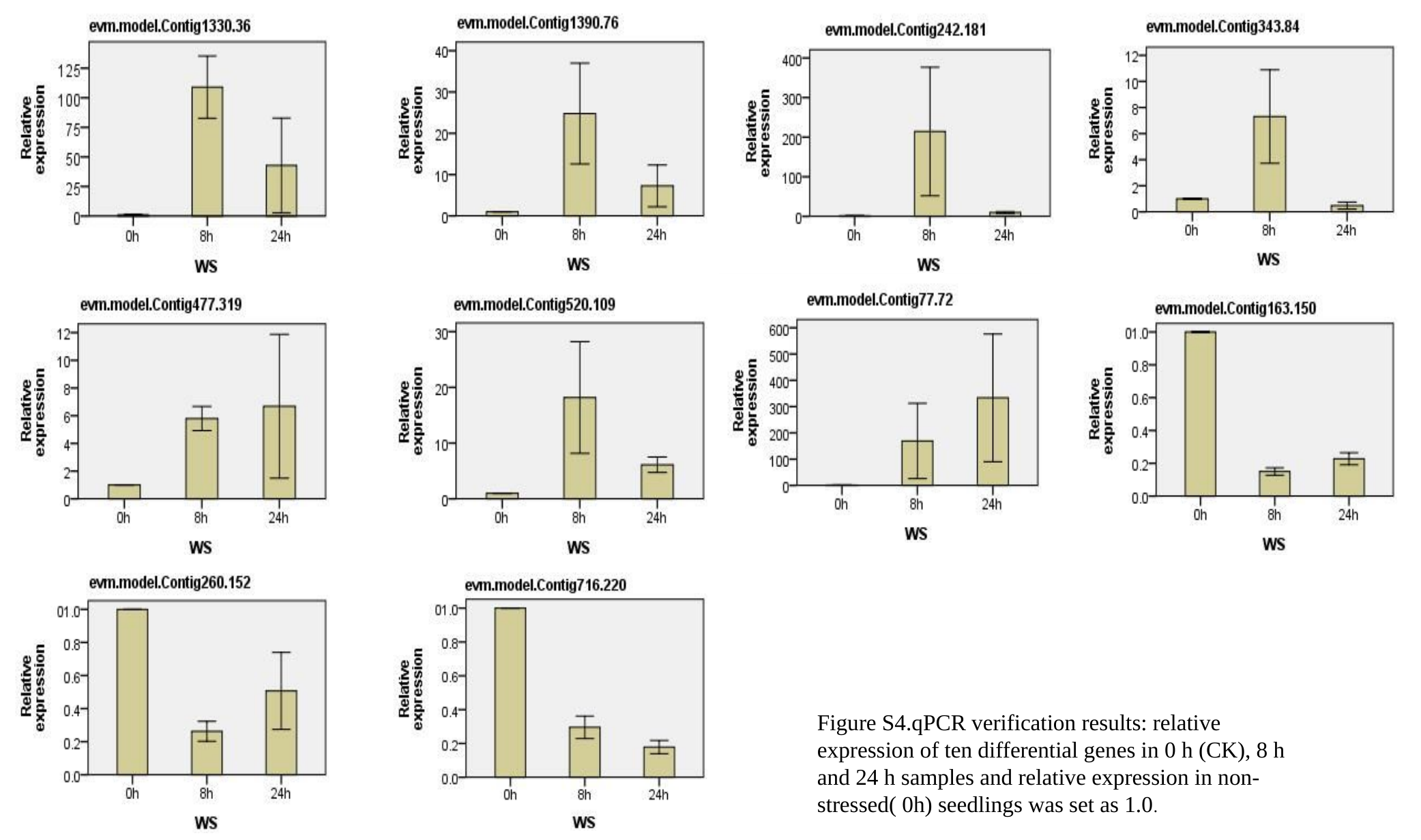

Figure S4.qPCR verification results: relative expression of ten differential genes in 0 h (CK), 8 h and 24 h samples and relative expression in non-stressed( 0h) seedlings was set as 1.0.

## Slide 5
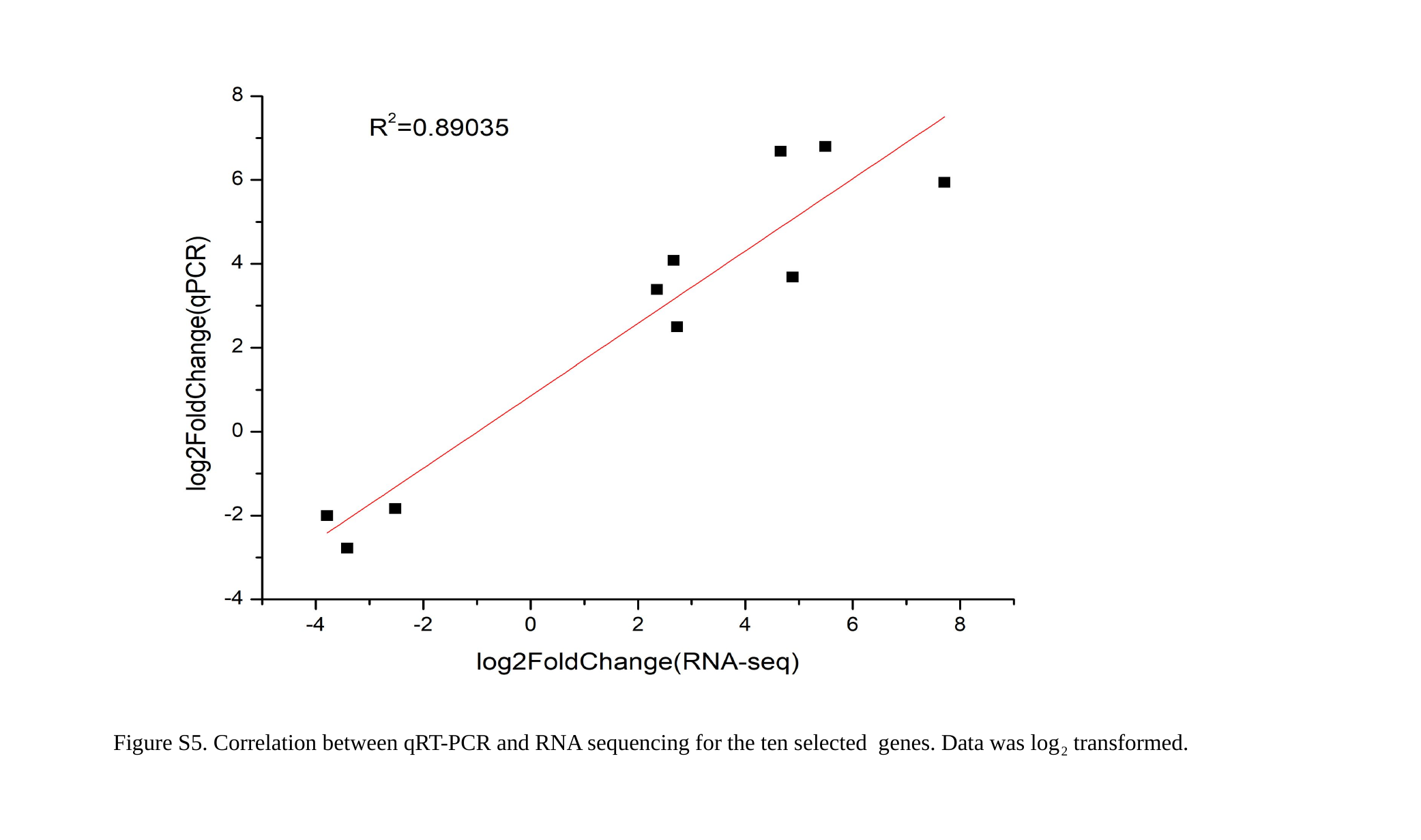

Figure S5. Correlation between qRT-PCR and RNA sequencing for the ten selected genes. Data was log2 transformed.
